# Factors associated with prevention practices against COVID-19 in the Peruvian population: Disparities between rural and urban areas

**DOI:** 10.1371/journal.pone.0267625

**Published:** 2022-05-10

**Authors:** Daniel Fernandez-Guzman, David R. Soriano-Moreno, Fabricio Ccami-Bernal, Randy Velasquez-Fernandez, Noelia Morocho-Alburqueque, Abraham De-Los-Rios-Pinto, Naomi Coba-Villan, Angelica Diaz-Corrales, Antony Pinedo-Soria, Pamela Grados-Espinoza, Wendy Nieto-Gutierrez

**Affiliations:** 1 Grupo Peruano de Investigación Epidemiológica, Unidad para la Generación y Síntesis de Evidencias en Salud, Universidad San Ignacio de Loyola, Lima, Perú; 2 Escuela Profesional de Medicina Humana, Universidad Nacional de San Antonio Abad del Cusco, Cusco, Perú; 3 Unidad de Investigación Clínica y Epidemiológica, Escuela de Medicina, Universidad Peruana Unión, Lima, Peru; 4 Universidad Nacional de San Agustín de Arequipa, Arequipa, Peru; 5 Facultad de Medicina, Universidad Nacional Mayor de San Marcos, Lima, Peru; 6 Escuela profesional de Medicina Humana, Facultad de Ciencias de la Salud, Universidad Nacional de Piura, Piura, Castilla, Peru; 7 Facultad de Medicina Humana, Universidad Nacional de Cajamarca, Cajamarca, Peru; 8 Facultad de Medicina Humana, Universidad de San Martin de Porres, Chiclayo, Peru; 9 Facultad de Medicina Humana, Universidad Nacional de San Martín, Tarapoto, Peru; 10 Facultad de Medicina Humana, Universidad Nacional Daniel Alcides Carrión, Cerro de Pasco, Peru; 11 Unidad de Investigación para la Generación de Síntesis de Evidencia en Salud, Universidad San Ignacio de Loyola, Lima, Peru; University of Arkansas for Medical Sciences, UNITED STATES

## Abstract

**Objective:**

To determine the factors associated with prevention practices against COVID-19 in the Peruvian population according to rural vs. urban locations.

**Methods:**

Analytical cross-sectional study, secondary analysis based on a previously collected database. A sample of individuals over 18 years of age, residing in Peru and with no history of COVID-19was evaluated. Factors associated with prevention practices were evaluated using Poisson regressions with variance adjustment by region cluster and stratified by rurality.

**Results:**

Of 3231 participants included, 2741 (84.8%) were from urban areas and 490 (15.2%) from rural areas. The frequency of good prevention practices against COVID-19 was 27.8% in our total sample. In urban areas the frequency of good prevention practices was 28.8% and in rural areas it was 22.5%. Factors associated with prevention practices against COVID-19 in both urban and rural areas were male sex (urban: aPR 0.64, 95%CI 0.55–0.75; rural: aPR 0.66, 95%CI 0.54–0.80) and self-considering adequately carrying out prevention practices (urban: aPR 2.48, 95%CI 2.13–2.89; rural: aPR 2.70, 95%CI 2.27–3.19).

**Conclusion:**

The frequency of good prevention practices against COVID-19 was less than 30% in both urban and rural areas. There are differences in the factors associated with good preventive practice against COVID-19. Only sex and considering that preventive measures were adequately carried out were associated with good prevention practices in both areas. In view of this, prevention measures should be promoted taking into account cultural principles and considering geographical location in the face of present and future outbreaks or pandemics.

## Introduction

The COVID-19 pandemic has been registering a progressive increase in the number of cases [1], causing more than 272 million infections and at least 5 million deaths worldwide by December 16, 2021 [2]. It has affected Latin American countries to a greater extent, such as Peru, where it has generated more than 2.2 million cases and at least 201 thousand deaths [3], making it the country with the highest mortality rate per million inhabitants worldwide up to December 16, 2021 [2].

Consequently, the Peruvian government, in an attempt to curb the transmission of the virus, as in other countries, implemented community containment measures [4], which complemented quarantine and social immobilization [5, 6], such as the mandatory use of masks, social distancing, and frequent hand washing [7]. However, adherence among residents to these new prevention measures was inadequate [8], leading to an increase in the incidence and mortality of COVID-19 within the country [9].

Different studies reported that poor prevention practices were associated with populations’ sociodemographic characteristics and knowledge about COVID-19 [10, 11]. These practices may vary according to rurality [12], due to the fact that in rural areas, there is less purchasing power and less access to health and social services [13, 14]. In addition, in rural areas, sociocultural factors, greater informal commercial activity and larger households could modify compliance with preventive measures compared to urban areas [9], since these characteristics could condition a greater need to obtain economic income during restrictive measures [15, 16].

Peru’s healthcare system has been fighting the pandemic but with limited results, one of the obstacles being the lack of local solutions considering the geographic diversity and characteristics of its population [17]. However, although there are studies in other countries on the factors associated with prevention practices with emphasis on the differences between rural and urban areas [18–20], the present study is the first to be carried out during the first wave of cases in Peru. Therefore, our objective was to determine the factors associated with prevention practices against COVID-19 in the Peruvian population according to rurality. This data could serve to provide health officials and policy makers with pieces of evidence that will contribute to the development of strategies and policies for the control of present and future outbreaks, with special emphasis on the characteristics and factors of the populations according to their rurality.

## Materials and methods

### Study design, setting and participants

We conducted an analytical cross-sectional study using secondary data from a study that evaluated prevention and control practices on a non-probabilistic sample of Peruvians over 18 years of age from the 24 regions of Peru, and that has been previously reported [21].

For the present study, only those participants who reported not having history of COVID-19 were included (3231 of 3630). Of the 3231 participants included, none had missing data on the variables of interest.

### Survey

A virtual questionnaire was designed and validated for the initial study (Cronbach’s alpha of 0.70 for the general survey) [21], which was structured in three sections: 1) sociodemographic characteristics; 2) perceptions and prevention practices of persons who did not have history of COVID-19 and 3) perceptions and measures taken to control the transmission of persons with history of COVID-19. For the present study, only the variables included in the first and second sections of the questionnaire were analyzed (S1 File).

### Outcome: “Prevention practices against COVID-19 infection”

The outcome of interest was obtained by evaluating 13 items that addressed compliance with prevention measures recommended by the Peruvian Ministry of Health and the conduction of activities that increased the risk of SARS-CoV-2 infection [7]. Each item consisted of a question that measured the frequency of a specific practice, which response options were structured using a Likert scale ranging from 1 "never" to 5 "always". Items that corresponded to negative questions were rated with an inverse score.

The prevention practices variable was constructed from the sum of the scores of the 13 variables with a minimum score of 13 and a maximum score of 65. Prevention practices were classified as good when the score was greater than or equal to 52 (upper quartile with a majority of answers in the categories: often and always), and as poor when the score was less than 52 (lower quartiles with a majority of answers in the categories: never, rarely, and sometimes).

### Other variables

Other variables were evaluated, such as sex (female, male), age (youth [18 to 29 years old], young adult [30 to 59 years old] or older adult [over 59 years old]), marital status (single, married or cohabiting), level of education (high school or less, higher education), region (coast, highlands, jungle), rurality (urban and rural), employment status(employed, unemployed), health insurance (none, Seguro Integral de Salud [SIS], Seguro Social de Salud [EsSalud], other [private insurance, insurance for the police and armed forces, etc.]). With respect to health insurance, the SIS is an insurance mainly focused on people living in poverty and extreme poverty, and also includes a small percentage of entrepreneurs, microenterprises and independent workers, while EsSalud is the contributory health insurance mainly aimed at dependent workers. Other variables considered were: socioeconomic level (low and medium-high; following the Graffar-Méndez stratification [22]), Presence of a health professional in the family (no, health science student, health professional), source from which you acquire information about COVID-19 (social networks, press media, information provided by physicians or scientific research [medical information], other), comorbidities for COVID-19 (no, yes), family member with comorbidity for COVID-19 (no, yes), family member diagnosed with COVID-19 (no, yes), relative deceased from COVID-19 (no, yes), use of medicine for COVID-19 prevention (no, yes), use of chlorine dioxide for COVID-19 prevention (no, yes), use of plants for COVID-19 prevention (no, yes), and some perceptions about the disease (if you consider as none, little and a lot). Government assistance was also considered as a variable, since the Peruvian government approved the distribution of economic bonuses to poor families.

Rurality was defined as "The territory with few inhabitants and few buildings where the main economic activity of the area is agriculture, cattle raising, fruit growing, horticulture, etc.". This concept was provided as a qualitative note in the survey question "Currently, do you live in a rural area?".

### Data entry, processing and analysis

Statistical analysis was performed with the Stata v.16 program. A stratified analysis was performed according to rurality (urban and rural) for both descriptive and inferential analysis. Absolute and relative frequencies were estimated for categorical variables and measures of central tendency and dispersion for numerical variables. The chi-square test was used to compare the proportions between groups.

Poisson regression was used to evaluate the associated factors, taking the region of origin as the cluster variable, thus obtaining prevalence ratios (PR) and their respective 95% confidence intervals (95%CI). For the multiple regression analysis, only those variables that were significant (p<0.05) in the bivariate analysis within each rurality stratum were included. Finally, the collinearity of the multivariate regression model was assessed using the variance inflation factor.

### Ethical considerations

The protocol of the present study was evaluated and approved by the institutional ethics committee of the Universidad Peruana Unión (Code: 2020-CEUPeU-00020) and registered in the Health Research Projects platform of the National Institute of Health, according to the Peruvian regulations for research on topics related to COVID-19 [23]. All of the participants provided their written consent for participating in this study before conducting the survey. The database did not include any variable that could potentially identify the participants.

## Results

The primary study evaluated a total of 3630 participants, of which 399 were excluded because they had a history of COVID-19, obtaining a final sample of 3231 respondents for the present analysis (84.8% from urban areas vs. 15.2% from rural areas).

Of the 2741 respondents from urban areas, 83.8% were youth, 62.9% were female, 75.6% had higher education, and 77.5% had a medium-high socioeconomic level. On the other hand, of the 490 respondents from rural areas, 89.0% were youth (18–29 years), 61.6% were female, 78.6% had higher education, and 52.2% had a medium-high socioeconomic level (Table 1).

**Table 1:** Sociodemographic characteristics of study participants, according to rurality strata and prevention practices (N = 3231).

Variables		Prevention practices							
		Urban				Rural			
		Total	Poor practices	Good practices	*p-value* *	Total	Poor practices	Good practices	*p-value* *
		N = 2741	N = 1953 (71.25%)	N = 788 (28.75%)		N = 490	N = 380 (77.55%)	N = 110 (22.45%)	
**Age**									
	Youth (18 to 29 years old)	2297 (83.8)	1663 (72.4)	634 (27.6)	**0.003**	436 (89.0)	337 (77.3)	99 (22.7)	0.698
	Young adult and older adults (> 30 years old)	444 (16.2)	290 (65.3)	154 (34.7)		54 (11.0)	43 (79.6)	11 (20.4)	
**Gender**									
	Female	1725 (62.9)	1147 (66.5)	578 (33.5)	**<0.001**	302 (61.6)	223 (73.8)	79 (26.2)	**0.013**
	Male	1016 (37.1)	806 (79.3)	210 (20.7)		188 (38.4)	157 (83.5)	31 (16.5)	
**Marital status**									
	Single	2393 (87.3)	1722 (72.0)	671 (28.0)	**0.032**	439 (89.6)	341 (77.7)	98 (22.3)	0.845
	Married or cohabiting	348 (12.7)	231 (66.4)	117 (33.6)		51 (10.4)	39 (76.5)	12 (23.5)	
**Grade of education**									
	High school or less	668 (24.4)	498 (74.6)	170 (25.5)	**0.030**	105 (21.4)	87 (82.9)	18 (17.1)	0.142
	Higher (technical or university)	2073 (75.6)	1455 (70.2)	618 (29.8)		385 (78.6)	293 (76.1)	92 (23.9)	
**Region**									
	Coast	1428 (52.1)	999 (70.0)	429 (30.0)	**<0.001**	153 (31.2)	116 (75.8)	37 (24.2)	0.055
	Highlands	1079 (39.4)	737 (68.3)	342 (31.7)		290 (59.2)	221 (76.2)	69 (23.8)	
	Jungle	234 (8.5)	217 (92.7)	17 (7.3)		47 (9.6)	43 (91.5)	4 (8.5)	
**Employment status**									
	No	2102 (76.7)	1500 (71.4)	602 (28.6)	0.819	407 (83.1)	314 (77.2)	93 (22.9)	0.637
	Yes	639 (23.3)	453 (70.9)	186 (29.1)		83 (16.9)	66 (79.5)	17 (20.5)	
**Health insurance**									
	None	835 (30.5)	590 (70.7)	245 (29.3)	0.635	139 (28.4)	108 (77.7)	31 (22.3)	0.594
	SIS	954 (34.8)	679 (71.2)	275 (28.8)		250 (51.0)	197 (78.8)	53 (21.2)	
	EsSalud	568 (20.7)	400 (70.4)	168 (29.6)		77 (15.7)	59 (76.6)	18 (23.4)	
	Other	384 (14.0)	284 (74.0)	100 (26.0)		24 (4.9)	16 (66.7)	8 (33.3)	
**Socioeconomic level**									
	Low	617 (22.5)	450 (72.9)	167 (27.1)	0.294	234 (47.8)	184 (78.6)	50 (21.4)	0.583
	Medium-High	2124 (77.5)	1503 (70.8)	621 (29.2)		256 (52.2)	196 (76.6)	60 (23.4)	
**Government assistance**									
	No	1977 (72.1)	1378 (69.7)	599 (30.3)	**0.004**	262 (53.5)	198 (75.6)	64 (24.4)	0.261
	Yes	764 (27.9)	575 (75.3)	189 (24.7)		228 (46.5)	182 (79.8)	46 (20.2)	
**Presence of a health professional in the family**									
	No	1017 (37.1)	736 (72.4)	281 (27.6)	0.387	240 (49.0)	184 (76.7)	56 (23.3)	0.825
	Health science student	438 (16.0)	317 (72.4)	121 (27.6)		80 (16.3)	64 (80.0)	16 (20.0)	
	Health professional	1286 (46.9)	900 (70.0)	386 (30.0)		170 (34.7)	132 (77.7)	38 (22.4)	

* p-value was calculated to compare differences between poor/good prevention practices according to participant characteristics. Calculated by Chi-square test of independence. p-values <0.05 are in bold.

Good prevention practices against COVID-19 were present in 27.8% of the total sample; 28.8% and 22.5% in urban and rural areas, respectively. In the bivariate analysis, in the urban area, a higher frequency (p<0.05) of good prevention practices was observed in young adults and older adults, female sex, married or cohabiting individuals, individuals with higher education, individuals from the highlands. On the other hand, in the rural area, a higher frequency (p<0.05) of good prevention practices was reported in the female sex (Table 1).

Regarding epidemiological characteristics, the source of information about COVID-19 was significantly associated with prevention practices in both sectors (p<0.05). But, having a family member with COVID-19 within the household and having used medications to prevent COVID-19 were associated with prevention practices only in urban areas (Table 2).

**Table 2:** Epidemiological characteristics of study participants, according to rurality strata and prevention practices (N = 3231).

Variables		Prevention practices							
		Urban				Rural			
		Total	Poor practices	Good practices	*p-value* *	Total	Poor practices	Good practices	*p-value* *
		N = 2741	N = 1953 (71.25%)	N = 788 (28.75%)		N = 490	N = 380 (77.55%)	N = 110 (22.45%)	
**Source where you acquire information about COVID-19**									
	Social networks	913 (33.3)	694 (76.0)	219 (24.0)	**<0.001**	185 (37.8)	152 (82.2)	33 (17.8)	**0.047**
	Press media	970 (35.4)	683 (70.4)	287 (29.6)		203 (41.4)	146 (71.9)	57 (28.1)	
	Medical information	766 (28.0)	497 (64.9)	269 (35.1)		81 (16.5)	63 (77.8)	18 (22.2)	
	Others	92 (3.4)	79 (85.9)	13 (14.1)		21 (4.3)	19 (90.5)	2 (9.5)	
**Comorbidities for COVID-19**									
	No	2338 (85.3)	1671 (71.5)	667 (28.5)	0.540	419 (85.5)	326 (77.8)	93 (22.2)	0.744
	Yes	403 (14.7)	282 (70.0)	121 (30.0)		71 (14.5)	54 (76.1)	17 (23.9)	
**Family member with comorbidity for COVID-19**									
	No	1316 (48.0)	955 (72.6)	361 (27.4)	0.143	271 (55.3)	206 (76.0)	65 (24.0)	0.365
	Yes	1425 (52.0)	998 (70.0)	427 (30.0)		219 (44.7)	174 (79.5)	45 (20.6)	
**Family member with COVID-19 diagnosis**									
	No	2031 (74.1)	1417 (69.8)	614 (30.2)	**0.004**	366 (74.7)	281 (76.8)	85 (23.2)	0.480
	Yes	710 (25.9)	536 (75.5)	174 (24.5)		124 (25.3)	99 (79.8)	25 (20.2)	
**Family member deceased from COVID-19**									
	No	2704 (98.7)	1922 (71.1)	782 (28.9)	0.090	484 (98.8)	375 (77.5)	109 (22.5)	0.733
	Yes	37 (1.4)	31 (83.8)	6 (16.2)		6 (1.2)	5 (83.3)	1 (16.7)	
**Use of medication for the prevention of COVID-19**									
	No	2166 (79.0)	1516 (70)	650 (30.0)	**0.005**	351 (71.6)	278 (79.2)	73 (20.8)	0.164
	Yes	575 (21.0)	437 (76.0)	138 (24.0)		139 (28.4)	102 (73.4)	37 (26.6)	
**Use of chlorine dioxide for the prevention of COVID-19**									
	No	2550 (93.0)	1808 (70.9)	742 (29.1)	0.140	422 (86.1)	323 (76.5)	99 (23.5)	0.182
	Yes	191 (7.0)	145 (75.9)	46 (24.1)		68 (13.9)	57 (83.8)	11 (16.2)	
**Use of medicinal plants for the prevention of COVID-19**									
	No	1155 (42.1)	833 (72.1)	322 (27.9)	0.391	148 (30.2)	118 (79.7)	30 (20.3)	0.447
	Yes	1586 (57.9)	1120 (70.6)	466 (29.4)		342 (69.8)	262 (76.6)	80 (23.4)	

* p-value was calculated to compare differences between poor/good prevention practices according to participant characteristics. Calculated by Chi-square test of independence. p-values <0.05 are in bold.

Regarding perceptions of the COVID-19 pandemic, a higher proportion of poor prevention practices was observed, with a statistically significant difference (p<0.05) in both areas (urban and rural), in those volunteers who considered "Not at all or a little" for adequately carrying out their prevention practices against COVID-19 and the use of the mask. While considering COVID-19 as " Not at all or a little" dangerous or deadly, it was associated with a higher proportion of poor prevention practices in the urban area (Table 3).

**Table 3:** Perceptions on COVID-19 of study participants, according to rurality strata and prevention practices (N = 3231).

Variables		Prevention practices							
		Urban				Rural			
		Total	Poor practices	Good practices	*p-value* *	Total	Poor practices	Good practices	*p-value* *
		N = 2741	N = 1953 (71.25%)	N = 788 (28.75%)		N = 490	N = 380 (77.55%)	N = 110 (22.45%)	
**Do you consider that COVID-19 has had a negative influence on your life?**									
	Not at all or a little	1185 (43.2)	862 (72.7)	323 (27.3)	0.132	198 (40.4)	159 (80.3)	39 (19.7)	0.229
	A lot	1556 (56.8)	1091 (70.1)	465 (29.9)		292 (59.6)	221 (75.7)	71 (24.3)	
**Do you consider that preventive measures against COVID-19 are practiced in your community?**									
	Not at all or a little	1438 (52.5)	1344 (71.9)	524 (28.1)	0.238	350 (71.4)	269 (78.0)	76 (22.0)	0.538
	A lot	1303 (47.5)	609 (69.8)	264 (30.2)		140 (28.6)	111 (76.6)	34 (23.5)	
**Do you consider that you are at increased risk of contracting COVID-19?**									
	Not at all or a little	2043 (74.5)	1455 (71.2)	588 (28.8)	0.949	376 (76.7)	295 (78.5)	81 (21.5)	0.382
	A lot	698 (25.5)	498 (71.4)	200 (28.7)		114 (23.3)	85 (74.6)	29 (25.4)	
**Do you consider your family to be at an increased risk of getting COVID-19?**									
	Not at all or a little	1472 (53.7)	1046 (71.1)	426 (28.9)	0.811	292 (59.6)	228 (78.1)	64 (21.9)	0.732
	A lot	1269 (46.3)	907 (71.5)	362 (28.5)		198 (40.4)	152 (76.8)	46 (23.2)	
**Do you consider COVID-19 to be a dangerous and deadly disease?**									
	Not at all or a little	623 (22.7)	476 (76.4)	147 (23.6)	**0.001**	127 (25.9)	105 (82.7)	22 (17.3)	0.108
	A lot	2118 (77.3)	1477 (69.7)	641 (30.3)		363 (74.1)	275 (75.8)	88 (24.2)	
**Do you consider that you adequately carry out preventive measures against COVID-19?**									
	Not at all or a little	804 (29.3)	700 (87.1)	104 (12.9)	**<0.001**	184 (37.6)	166 (90.2)	18 (9.8)	**<0.001**
	A lot	1937 (70.7)	1253 (64.7)	684 (35.3)		306 (62.5)	214 (69.9)	92 (30.1)	
**Do you consider that the use of the mask protects you from contracting COVID-19?**									
	Not at all or a little	650 (23.7)	511 (78.6)	139 (21.4)	**<0.001**	184 (37.6)	157 (85.3)	27 (14.7)	**0.001**
	A lot	2091 76.3)	1442 (69.0)	649 (31.0)		306 (62.5)	223 (72.9)	83 (27.1)	
**Do you think there are many cases of COVID-19 in your community?**									
	Not at all or a little	1438 (52.5)	1047 (72.8)	391 (27.2)	0.058	350 (71.4)	274 (78.3)	76 (21.7)	0.538
	A lot	1303 (47.5)	906 (69.5)	397 (30.5)		140 (28.6)	106 (75.7)	34 (24.3)	
**Do you consider that taking medicines, plants or other substances protects you from getting sick from COVID-19?**									
	Not at all or a little	2246 (81.9)	1593 (70.9)	653 (29.1)	0.423	364 (74.3)	286 (78.6)	78 (21.4)	0.358
	A lot	495 (18.1)	360 (72.7)	135 (27.3)		126 (25.7)	94 (74.6)	32 (25.4)	

* p-value was calculated to compare differences between poor/good prevention practices according to participant perceptions. Calculated by Chi-square test of independence. p-values <0.05 are in bold.

In the urban areas, the multivariate analysis results suggested that the factors associated with good prevention practices were being young adults or older adults (aPR: 1.23; 95%CI: 1.07–1.41), having a higher level of education (aPR: 1.13; 95%CI: 1.06–1.21), acquiring information from the press media (aPR: 1.19; 95%CI: 1.16–1.22) and by physicians (aPR: 1.39; 95%CI: 1.32–1.47), having consumed medicinal plants for COVID-19 prevention (aPR: 1.05; 95%CI: 1.01–1.10), and considering that prevention measures were adequately carried out (aPR: 2.48, 95%CI: 2.13–2.89). On the other hand, factors associated with poor prevention practices were, being male (aPR: 0.64, 95%CI: 0.55–0.75), having EsSalud insurance (aPR: 0.84; 95%CI: 0.77–0.92) or other non-state insurance (aPR: 0.79; 95%CI: 0.64–0.98), having received some type of government assistance in the face of the pandemic (aPR: 0.86; 95%CI: 0.80–0.92), and having a family member diagnosed with COVID-19 (aPR: 0.82; 95%CI: 0.72–0.94) (Table 4).

**Table 4:** Factors associated with prevention practices for COVID-19 infection in urban areas.

Characteristics		Crude Model			Adjusted Model		
		cPR	95%CI	p	aPR	95%CI	p
**Age**							
	Youth (18 to 29 years old)	Ref.			Ref.		
	Young adult and older adults (> 30 years old)	1.26	1.08–1.46	**0.002**	1.23	1.07–1.41	**0.004**
**Gender**							
	Female	Ref.			Ref.		
	Male	0.62	0.51–0.75	**<0.001**	0.64	0.55–0.75	**<0.001**
**Grade of education**							
	High school or less	Ref.			Ref.		
	Higher (technical or university)	1.17	1.03–1.33	**0.013**	1.13	1.06–1.21	**<0.001**
**Health insurance**							
	None	Ref.			Ref.		
	SIS	0.98	0.91–1.06	0.655	0.97	0.92–1.02	0.262
	EsSalud	1.01	0.92–1.10	0.856	0.84	0.77–0.92	**<0.001**
	Other	0.89	0.79–0.99	**0.047**	0.79	0.64–0.98	**0.031**
**Government assistance**							
	No	Ref.			Ref.		
	Yes	0.82	0.78–0.86	**<0.001**	0.86	0.80–0.92	**<0.001**
**Source where you acquire information about COVID-19**							
	Social networks	Ref.			Ref.		
	Press media	1.23	1.20–1.27	**<0.001**	1.19	1.16–1.22	**<0.001**
	Medical information	1.46	1.36–1.57	**<0.001**	1.39	1.32–1.47	**<0.001**
	Others	0.59	0.22–1.58	0.294	0.65	0.26–1.62	0.354
**Family member with COVID-19 diagnosis**							
	No	Ref.			Ref.		
	Yes	0.81	0.68–0.97	**0.024**	0.82	0.72–0.94	**0.005**
**Family member deceased from COVID-19**							
	No	Ref.			Ref.		
	Yes	0.56	0.34–0.91	**0.020**	0.69	0.47–1.03	0.067
**Use of chlorine dioxide for the prevention of COVID-19**							
	No	Ref.			Ref.		
	Yes	0.83	0.73–0.94	**0.005**	0.97	0.89–1.06	0.498
**Use of medicinal plants for the prevention of COVID-19**							
	No	Ref.			Ref.		
	Yes	1.05	1.01–1.10	**0.027**	1.10	1.05–1.16	**<0.001**
**Do you consider that COVID-19 has had a negative influence on your life?**							
	Not at all or a little	Ref.			Ref.		
	A lot	1.10	1.06–1.14	**<0.001**	1.02	0.97–1.08	0.450
**Do you consider COVID-19 to be a dangerous and deadly disease?**							
	Not at all or a little	Ref.			Ref.		
	A lot	1.28	1.26–1.31	**<0.001**	1.08	0.99–1.17	0.101
**Do you consider that you adequately carry out preventive measures against COVID-19?**							
	Not at all or a little	Ref.			Ref.		
	A lot	2.73	2.36–3.15	**<0.001**	2.48	2.13–2.89	**<0.001**
**Do you consider that the use of the mask protects you from getting COVID-19?**							
	Not at all or a little	Ref.			Ref.		
	A lot	1.45	1.22–1.72	**<0.001**	1.06	0.92–1.22	0.458

cPR: crude Prevalence Ratio; aPR: adjusted Prevalence Ratio.

Prevalence ratios and confidence intervals were calculated considering statistical criterion (p-value <0.05 in the bivariate regression). p-values <0.05 are in bold.

Collinearity was evaluated using the variance inflation factor, resulting in a value of less than 5 for all the variables analyzed.

In the rural areas, the multivariate analysis results indicated that the factors associated with good prevention practices were considering that prevention measures were adequately carried out (aPR: 2.70, 95%CI: 221 2.27–3.19). On the other hand, factors associated with poor prevention practices, were being male (aPR: 0.66, 95%CI: 0.54–0.80), being a health professional (aPR: 0.91; 95%CI: 0.85–0.98), being informed by friends and family (aPR: 0.62; 95%CI: 0.40–0.97), and having a family member with comorbidity for COVID-19 (aPR: 0.84; 95%CI: 0.77–0.91) (Table 5).

**Table 5:** Factors associated with prevention practices for COVID-19 infection in rural areas.

Characteristics		Crude Model			Adjusted Model		
		cPR	95%CI	p	aPR	95%CI	p
**Gender**							
	Female	Ref.			Ref.		
	Male	0.63	0.49–0.82	**0.001**	0.66	0.54–0.80	**<0.001**
**Presence of a health professional in the family**							
	No	Ref.			Ref.		
	Health science student	0.86	0.75–0.98	**0.023**	0.82	0.64–1.07	0.140
	Health professional	0.96	0.88–1.04	0.297	0.91	0.85–0.98	**0.016**
**Source where you acquire information about COVID-19**							
	Social networks	Ref.			Ref.		
	Press media	1.57	1.20–2.07	**0.001**	1.42	1.00–2.00	**0.049**
	Medical information	1.25	0.92–1.69	0.161	1.13	0.77–1.65	0.537
	Others	0.53	0.30–0.96	**0.036**	0.62	0.40–0.97	**0.037**
**Family member with comorbidity for COVID-19**							
	No	Ref.			Ref.		
	Yes	0.86	0.81–0.91	**<0.001**	0.84	0.77–0.91	**<0.001**
**Do you consider that COVID-19 has had a negative influence on your life?**							
	Not at all or a little	Ref.			Ref.		
	A lot	1.23	1.04–1.46	**0.013**	1.05	0.88–1.27	0.576
**Do you consider that you adequately carry out preventive measures against COVID-19?**							
	Not at all or a little	Ref.			Ref.		
	A lot	3.07	2.58–3.66	**<0.001**	2.70	2.27–3.19	**<0.001**
**Do you consider that the use of the mask protects you from getting COVID-19?**							
	Not at all or a little	Ref.			Ref.		
	A lot	1.85	1.36–2.51	**<0.001**	1.34	0.99–1.81	0.062
**Do you consider that taking medicines, plants or other substances protects you from getting sick from COVID-19?**							
	Not at all or a little	Ref.			Ref.		
	A lot	1.19	1.01–1.39	**0.039**	1.06	0.99–1.13	0.056

cPR: crude Prevalence Ratio; aPR: adjusted Prevalence Ratio.

Prevalence ratios and confidence intervals were calculated considering statistical criterion (p-value <0.05 in the bivariate regression). p-values <0.05 are in bold.

Collinearity was evaluated using the variance inflation factor, resulting in a value of less than 5 for all the variables analyzed.

## Discussion

Prevention practices represent the main way to combat and further stop COVID-19 transmission in all regions of the world [24, 25]. Full participation of the population is crucial for adherence and proper implementation [11]. In the present investigation, we included participants residing in urban and rural areas, with similar proportions in terms of age, sex, marital status, and educational level. It was found that gender, the source of information about COVID-19, and the perception of adequate prevention practices were associated in both areas studied. While the factors related to prevention practices only in urban areas were age group, education level, health insurance, Government assistance, having a family member diagnosed with COVID-19 and the use of medicinal plants to prevent COVID-19. Likewise, in rural areas, the presence of a health professional in the family and having a family member with comorbidities for COVID-19 were associated with prevention practices.

We found a frequency of good prevention practices of less than 30%, both in the urban and rural sectors. This phenomenon has already been observed in previous pandemics with similar characteristics in Peru, with less than 50% of participants reporting good prevention practices, which was attributed to an inadequate level of knowledge about the disease [26]. Likewise, although the percentages observed for prevention against COVID-19 in our sample were similar to those reported in Nigeria (36%), it was much lower than those identified in Bangladesh [27], Ethiopia [28], Uganda [29], and Palestine [30], which frequencies of good prevention practices ranged from 77 to 99%. This discrepancy may be due to the fact that these countries have recently faced epidemics of other highly infectious diseases such as Ebola, leading to the familiarization with similar measures to prevent infection [31].

On the other hand, a lower percentage of good practices was observed in the rural sector compared to the urban sector, probably because in our study the majority of respondents from rural areas reported perceiving COVID-19 as a disease that is not so dangerous or deadly and with a perception that it presents a minimal risk of infection. This scenario was also observed in previous studies conducted in the United States [27], and Bangladesh [12], where prevention attitudes in rural areas were lower than in urban areas. This observed state of little understanding of the harm caused by the virus in rural areas calls for greater emphasis on prevention strategies focused on areas of residence, where priority is given not only to teaching the necessary preventive measures, but also to disseminating information on the situation of COVID-19 infection, its possible complications, and the health situation in the country.

Among the most relevant associated factors in the urban sector, it was found that being a young adult or older adult was associated with a higher frequency of good prevention practices. This could be due to the fact that in this age group the probability of developing the severe form of COVID-19 was higher; therefore, it is to be expected that these people would be more careful and have better adherence to preventive measures [10, 27]. However, this association was not observed in the rural population, probably due to the lack of information provided in these communities, leading to misinformation about the populations at risk and the consequences of COVID-19 [12, 32].

Having a higher level of education was associated with a higher frequency of good prevention practices in the urban sector, which strengthens the idea that education would favor recognition of the importance of preventive measures [33]. However, despite the fact that the majority of rural respondents also had higher education, this association was not observed. Rural areas are characterized by highlighting cultural beliefs in their actions and thoughts, fostering distrust of those outside their area of residence [32]. Therefore, considering that the prevention measures were imposed at the national level and not by their local leaders, it is likely to have generated distrust about the certainty of the information and therefore possibly generated a questioning of the importance of complying with them [12]. It is also necessary to consider the limited access to health information in these areas [34], which could be affecting even those with higher education.

Furthermore, it was found that having a family member with COVID-19 in the household was associated with a lower frequency of prevention practices in the urban sector. Although no previous studies were found that evaluated this factor, we believe that this behavior of neglecting to prevent SARS-CoV-2 infection could be due to the humanitarian and economic crisis experienced in Peru [35]. This possibly forced people to continue going out to the streets to obtain economic support and continue to support their families, especially if they had a sick family member who needed medical care [36].

In view of the fact that the Peruvian people were experiencing a health, economic and humanitarian crisis, the Peruvian state decided to distribute economic support to the most economically vulnerable groups [37]. Our study found that receiving such support was associated with a lower frequency of good prevention practices in urban areas. It is important to take into account that this economic bonus had an amount of 760 Peruvian Soles and was distributed to the lowest economic strata [37]; however, the average cost of living exceeds the amount provided [38], which possibly implies that they had to continue working for a higher income to cover their minimum expenses [39], and consequently the neglect of prevention practices.

Regarding the associated factors in rural areas, we found that the presence of a health professional or health sciences student in the family were associated with a lower frequency of prevention practices. Previous studies have identified a lower level of knowledge about COVID-19 in rural areas [40], including a lack of concern by health workers in the implementation of preventive measures in these areas [41]. This, added to the feeling of security that could be generated the presence of a health professional or health sciences student in the family, believing that they have more knowledge and practices to protect themselves against the new virus [42], it is to be expected to find this seemingly paradoxical association.

In addition, it was found that having a family member with some comorbidity for COVID-19 was associated with a lower frequency of good prevention practices in rural areas. Due to Peruvian regulations, many of the people with comorbidity have been unable to perform face-to-face work [43], which in many cases has led to dismissal and loss of their jobs, influencing families to redistribute responsibilities for obtaining economic income [44]. In this case, it can be expected that in our rural population surveyed (mostly youth people) they have the obligation to make up for the lost economic income, exposing themselves more to the virus, to greater stress in obtaining income, and consequently less concern in carrying out the correct prevention practices [45].

Regarding the variables that were associated in both the urban and rural sectors, it was observed that being male was significantly associated with a lower frequency of good prevention practices. This was in agreement with the findings of studies in Bangladesh [27] and China [46], and was distinct from the findings in Cameroon [47]. It is reported that women tend to be more concerned about their health than men [48]. This is in addition to the fact that the social reality in Peru describes that men tend to be responsible for the support of their households [38], so it is likely that they are more concerned about earning income and consequently less concerned about preventive measures [49].

On the other hand, although information from medical sources or the press was found to be associated with a higher frequency of good practices in urban areas, this was not observed in rural areas. On the contrary, in rural areas, information obtained from friends and relatives was associated with a lower frequency. It is important to emphasize the importance of the use of reliable health information, prioritizing those provided by health personnel, especially in the current context where there has been an increase in false news regarding the disease and its prevention [50]. Although previous studies have identified that rural areas tend to prioritize the information provided by their community (leaders, neighbors, etc.) [32], the reason for this phenomenon is not well understood [51]. However, it is imperative to promote and implement dissemination strategies for the identification of false news, access to good sources of information, and punishment of those who disturb public health [52]; prioritizing the rural sector.

Finally, the use of substances such as medicines and chlorine dioxide were not significantly related to COVID-19 prevention practices in any sector. Although we expected this association, it has been reported that the use of preventive substances generates a false perception of safety and worse prevention practices [53]. We do not rule out the interference of selection bias in the underestimation of this association, so future studies should corroborate or reject the findings.

Our results suggest that there are different factors that influence preventive practices among rural and urban populations. However, it should be kept in mind that the source of information is a modifiable factor that influences prevention practices in both groups. Therefore, it is necessary, as a first point, the generation of information channels by the state where prevention practices are promoted with scientific evidence and highlighting the importance of continuing to perform them after vaccination.

The present study has certain limitations to be taken into account. Firstly, non-probabilistic sampling was used to enroll the participants, making it difficult to extrapolate the results. Also, because the survey was virtual and published via WhatsApp, Facebook and Instagram social networks and distributed through groups and direct messages to acquaintances, friends and family, it could have led to the population having similar characteristics to the authors and collaborators who distributed the survey. This is reflected in that the majority of the sample was predominantly youth, with higher education and living in an urban area. Additionally, because the survey was based on self-report, participants who had asymptomatic or mild disease may believe they did not have the disease and may have been included in the present study. Similarly, in the absence of direct information on geocoding or geolocation, the delimitation between place of residence (rural or urban) was influenced by the perception of the respondents.

Despite these limitations, this is the first study to evaluate the factors associated with prevention practices against COVID-19 disease in the Peruvian population. Likewise, a stratification by rurality was performed in order to better understand the differences between both realities. Finally, the sample size was considerable, involving a total of 3231 individuals.

## Conclusions

In conclusion, the frequency of good prevention practices against COVID-19 was less than 30% in both urban and rural areas. Being informed about COVID-19 through the media and considering that preventive measures were adequately carried out were associated with a higher frequency of good prevention practices in both rural and urban areas, while being male was associated with a lower frequency in both sectors. The stratification of the population by rurality reflects differences between sectors and the need for focused approaches in the health policies to be implemented. Preventive measures should be promoted and encouraged on an ongoing basis, taking into account the cultural principles of all Peruvians.

## Supporting information

S1 Data (DO)

S2 Data (DTA)

S1 File (DOCX)
