# Supplementary material for: Factors associated with prevention practices against COVID-19 in the Peruvian population: Disparities between rural and urban areas
Source: PLoS One. 2022 May 10;17(5):e0267625. doi: 10.1371/journal.pone.0267625 (PMC9089852; doi:10.1371/journal.pone.0267625)
Supplement: S1 File — (DOCX) [file pone.0267625.s003.docx]

**SUPPLEMENTARY MATERIAL 1: SURVEY**

**SECTION 1: GENERAL DATA**

1. How old are you?____ (in years old)

2. What is your gender?

- Woman
- Man

3. What is your marital status?

- Bachelor
- Married or cohabiting
- Divorced or Separated
- Widower

4. What degree of instruction do you have?

- Complete or incomplete primary
- Incomplete secondary
- Complete Secondary
- Non-superior technician
- Senior Technician
- University Superior

5. What is your employment status?

- Employee
- Unemployed

6. What kind of health insurance do you have?

- None
- SIS (MINSA)
- Social Security (EsSalud)
- Private clinic
- Armed forces or police insurance
- Other: ______________

7. Where do you live? (Department, example: Cusco) ____________________

8. Do you currently live in a rural area? (Consider as a rural area the territory with few inhabitants and few buildings where the main economic activity of the area is agriculture, cattle raising, fruit growing, horticulture, etc.)

- No
- Yes

9. Did your family receive any support from the government (government economic bonds)?

- No
- Yes

10.Do you have a family member inside your home working in a hospital?

- Yes, a family health professional
- Yes, I am a health professional
- No, no one in my family is a health professional
- No, but I have a family member who is a student in health sciences
- No, but I am a student in health sciences

11.What source of information do you use the most, to be aware of COVID 19?

- Social networks (Facebook, YouTube, Instagram, Twitter, etc.)
- information from friends, family or neighbors (including WhatsApp messages)
- Television or radio information
- Media (newspapers, magazines, etc.)
- Medical information (doctors, scientific journals, etc.)

12. Do you have any of the following health conditions?

- Diabetes mellitus
- High blood pressure (highbloodpressure)
- Obesity (Body Mass Index >=30)
- Chronic Renal Failure
- Asthma, cystic fibrosis, or COPD
- Immunosuppression (weakened immune system including Cancer)
- Serious heart condition (heart failure or history of heart attack)
- She is pregnant
- None

13.Does anyone in your household have any of the health conditions listed in the previous question or is there someone over the age of 65?

- No
- Yes

14.Were any members of your household diagnosed with COVID-19? (without considering you)

- No
- Yes

15. Were you diagnosed with COVID-19?

- No
- Yes

**SECTION 2: PRACTICES AND PERSPECTIVES ON COVID-19 (section only for those who did not report a history of COVID-19)**

22. How often... (Mark with an "**X**")

| INTERNSHIP | **NEVER** | **SELDOM** | **SOMETIMES** | **OFTEN** | **ALWAYS** |
| --- | --- | --- | --- | --- | --- |
| ... do you wear a mask when leaving home? |  |  |  |  |  |
| ... do you bring your hands unwashed to your eyes, nose or mouth? |  |  |  |  |  |
| ... covers your nose and mouth when sneezing or coughing? |  |  |  |  |  |
| ... do you wear a face mask when leaving home? |  |  |  |  |  |
| ... keeps distance from others on the street (at least 1 m)? |  |  |  |  |  |
| ... is accompanied when going to the street? |  |  |  |  |  |
| ... do you wash your hands with water and soap when you return home? |  |  |  |  |  |
| ... disinfects surfaces of personal objects and places? |  |  |  |  |  |
| ... shake hands, bump fist or elbow with your friends? |  |  |  |  |  |
| ... disinfect your clothing when you get home? |  |  |  |  |  |
| ... disinfect your shoes when you get home? |  |  |  |  |  |
| ... disinfect your purchases when you get home? |  |  |  |  |  |
| ... disinfect your money when you get home? |  |  |  |  |  |

| 23. In the last 2 weeks. How many times... | **0** | **1** | **2** | **3** | **4** | **5** | **6** | **7** | **8** | **9** | **10** | **11** | **12** | **13** | **≥14** |
| --- | --- | --- | --- | --- | --- | --- | --- | --- | --- | --- | --- | --- | --- | --- | --- |
| ... have you changed or renewed your mask? |  |  |  |  |  |  |  |  |  |  |  |  |  |  |  |
| ... have you left home? |  |  |  |  |  |  |  |  |  |  |  |  |  |  |  |
| ... have you gone to markets, shops, shopping malls or banks? |  |  |  |  |  |  |  |  |  |  |  |  |  |  |  |
| ... have you attended religious, social and/or recreational activities? |  |  |  |  |  |  |  |  |  |  |  |  |  |  |  |
| ... have you boarded public transport (Combis, metros, train, etc.)? |  |  |  |  |  |  |  |  |  |  |  |  |  |  |  |
| ... have you used medications to prevent COVID-19? |  |  |  |  |  |  |  |  |  |  |  |  |  |  |  |
| ... have you used medicinal plants in order to prevent COVID-19? |  |  |  |  |  |  |  |  |  |  |  |  |  |  |  |
| ... Have you used chlorine dioxide in order to prevent COVID-19? |  |  |  |  |  |  |  |  |  |  |  |  |  |  |  |

24. What material do you most use your mask?

- Homemade mask of 1 or 2 layers
- Homemade mask from 3 to more layers
- Disposable surgical mask (purchased at pharmacy)
- Mask N-95
- Kn-95 Mask
- Respirator with filter
- Other: _________________

25. How long does it take to wash your hands?

- ____________ seconds

26. What medicines do you take exclusively to prevent COVID-19?

- None
- Ivermectin for human use
- Ivermectin for veterinary use
- Hydroxychloroquine
- Azithromycin or Clarithromycin
- Corticosteroids (dexamethasone, prednisone, etc.)
- Paracetamol
- NSAIDs (ibuprofen, naproxen, or diclofenac)
- Heparin or Warfarin (or another anticoagulant)
- Other: ______

27. What main source of information did you use for the use of these medicines?

- I didn't use medication
- Social networks (Facebook, YouTube, Instagram, Twitter, etc.)
- information from friends, family or neighbors (including WhatsApp messages)
- Television or radio information
- Media (newspapers, magazines, etc.)
- Medical information (doctors, scientific journals, etc.)

28. What medicinal plants do you use exclusively to prevent COVID 19? (

- None
- Eucalyptus
- Matico
- Mallow
- Plantain
- Quinine
- Lemon verbena
- Cypress
- Soursop Leaves
- Lemon Leaves
- Kion
- Bands
- Other: ____

29. What main source of information did you use for the use of these plants?

- I didn't use plants
- Social networks (Facebook, YouTube, Instagram, Twitter, etc.)
- information from friends, family or neighbors (including WhatsApp messages)
- Television or radio information
- Media (newspapers, magazines, etc.)
- Medical information (doctors, scientific journals, etc.)

30. As a preventive measure, what do you think about the use:

|  | It's effective, it does good | It's effective, it does little harm | Not effective, but it doesn't hurt | It is not effective and does harm | I am not informed of that issue |
| --- | --- | --- | --- | --- | --- |
| Ivermectin |  |  |  |  |  |
| Other medicines |  |  |  |  |  |
| Chlorine Dioxide |  |  |  |  |  |
| Medicinal plants |  |  |  |  |  |
| The possible COVID-19 vaccine |  |  |  |  |  |

31. To what extent do you consider...

|  | A lot | Little | Nothing |
| --- | --- | --- | --- |
| ... that COVID-19 has negatively influenced your life? |  |  |  |
| ... what are preventive measures against COVID-19 in your community? |  |  |  |
| ... that you are at higher risk of catching COVID-19? |  |  |  |
| ... that your family is exposed to an increased risk of catching COVID-19? |  |  |  |
| ... that COVID-19 is a dangerous and deadly disease? |  |  |  |
| ... that properly carries out the prevention measures against COVID-19? |  |  |  |
| ... that wearing a mask protects you from catching COVID-19? |  |  |  |
| ... that there are many cases of COVID-19 in your community? |  |  |  |
| ... that rapid tests are reliable? |  |  |  |
| ... that taking medicines, plants or other substances protect from getting sick from COVID-19? |  |  |  |
